# SEM and qRT-PCR revealed quercetin inhibits morphogenesis of *Aspergillus flavus* conidia via modulating calcineurin-Crz1 signalling pathway

**DOI:** 10.1080/21501203.2020.1711826

**Published:** 2020-01-13

**Authors:** Sonia K. Shishodia, Shraddha Tiwari, Shanu Hoda, Pooja Vijayaraghavan, Jata Shankar

**Affiliations:** a Genomics laboratory, Department of Biotechnology and Bioinformatics, Jaypee University of Information Technology, Solan, India; b Amity Institute of Biotechnology, Amity University Uttar Pradesh, Noida, India

**Keywords:** *Aspergillus flavus*, Scanning electron microscopy, qRT-PCR, Calcium signalling pathway, Quercetin

## Abstract

**Aspergillus flavus:**

exploits diverse mechanisms to survive during exposure to antifungal agents including morphogenesis. Germination of dormant conidia involves cascades of reactions integrated into the signalling pathway. This study documents the effect of phytochemical-quercetin on *A. flavus* during germination of conidia using scanning electron microscopy (SEM). Significant inhibition of conidial swelling of *A. flavus* in comparison to control was observed at 4 and 7 h Quantitative real-time PCR for genes from calcium signalling pathway and heat-shock proteins family showed up-regulation of heat shock (*Hsp70* and *Hsp90*) and calcium signalling pathway genes (calcium-transporting ATPase and calmodulin) in response to quercetin at initial 4 h in comparison to control sample whereas up-regulation of *Hsp70*, calcineurin and transcription factor *Crz1*, were observed in both the treated samples. Gene encoding for calcium-kinase, *cAMP, Rho-gdp, Plc* and *Pkc* showed a constitutively higher level of expression in quercetin-treated sample in comparison to control at both time points. These data showed a clear response from genes encoding calcineurin-Crz1 signalling pathways and may find its application in the screening of antifungal agents.

**Abbreviations:**

Hsp: Hear shock protein; MIC: Minimum Inhibitory Concentration; SEM: Scanning Electron Microscopy; qRT-PCR: Quantitative Real-Time Polymerase Chain Reaction

## Introduction

The morphological transition of dormant conidia of *Aspergillus flavus* to the mycelial stage is critical for it to be a successful pathogen. *A. flavus* conidia colonise on crops under the favourable condition and on successful germination produce carcinogenic aflatoxin. It exists in three forms *viz*. conidia, mycelia, and hyphae and metabolic adaptation are required for the isotropic growth of the conidia to germinate into the hyphae (Shankar et al. 2018). The anti-fungal agents available (echinocandins, azoles and amphotericin B) majorly target the fungal cell wall and cell membrane (McCarthy et al. 2017). The poor outcome or failure of antifungal agents are generally attributed to the development of drug-resistant clinical or environmental isolates (Shishodia et al. 2019). In fungi, complex signal transduction cascades regulating morphogenesis are important to adapt and survive under influence of antifungal drugs (Lengeler et al. 2000). Calcium signalling pathway has been widely studied in *C. albicans* and *S. cerevisiae* in response to various stress and found to play a major role in cell survival, morphogenesis and virulent activity (Cowen and Steinbach 2008; Liu et al. 2015a). It is noted that the cellular calcium signalling machinery interacts with many other signalling pathway and is conserved throughout the fungal system (Barrige et al. 2003). Also, calcium signalling is directly involved in lateral branching from subapical hyphal compartments separated from the tip by a septum, hence playing a vital role in fungal morphogenesis. Previous researches have shown that binding of Ca^2+^ with calmodulin activates calmodulin-dependent kinases and the calcineurin phosphatase. Ca^2+^ binding to the regulatory subunit of calcineurin (CnaB) allowing activation of the catalytic subunit (CnaA) (Joseph and Means 2002). CnaA dephosphorylates transcription factor, crzA, to activate nuclear genes controlling processes such as cell-wall remodelling, polarity and conidiophore development and to respond against various stress signals (Yoshimoto et al. 2002; Boyce et al. 2015). Numerous studies have emphasised the role of Ca^2+^ in polarised tip growth in filamentous fungi. Tip-high gradients of total and free cytoplasmic Ca^2+^ have been measured in the pollen tube and growing fungal hyphae (Torralba and Heath 2001). Along with calcium signalling, heat shock proteins (Hsps) also impart their role in fungal morphogenesis. Heat shock proteins, being conserved in eukaryotes and its important role in morphogenesis and virulence in response to stress have been found attractive for antifungal studies (Gong et al. 2017). In addition, Hsps interacts with other cellular signalling pathways in order to control various physiological activities and virulence in fungi in response to traditional antifungal drugs (Singh et al. 2009; Gong et al. 2017). Previously it has been shown that Hsp90 and Hsp70 are the two major heat-shock proteins which can alone or together play a major role in morphogenesis and filamentation in *Aspergilli* (Tiwari et al. 2015). It has also been reported that Hsp90 is linked with calcium signalling as it regulates calcineurin (Mkc1) in the maintenance of the integrity of cell wall with the help of Hsp70 (LaFayette et al. 2010). Previously it has been shown that Hsp90-calcineurin pathway control conidiation in *A. fumigatus* and *A. nidulans*. However, Hsp90-calcineurin pathway inhibition resulted in hyphal growth impairment, cell-wall inhibition and defect in sporulation in *A. fumigatus* (Juvvadi et al. 2014). In addition, calcineurin helps Hsp90 in maintaining environmental changes by regulating dimorphism but not proliferation (Tiwari et al. 2015; Lamoth et al. 2016).

Anti-fungal studies have been approached towards some novel, environmental friendly compounds, which are basically plant derivatives such as alkaloids, flavanoids, tannins etc. (Arif et al. 2009). Phytochemicals against *A. flavus* showed that quercetin is a potential plant extract having both anti-*Aspergillus* and antiaflatoxigenic properties using MTT assay analysis (Zhou et al. 2015; Tiwari et al. 2017). Quercetin, a polyphenolic flavanoid have shown to exhibit oxidative stress in various *Aspergillus* species as well anti-fungal activities against *A. flavus and C. abicans* (Tempesti et al. 2012; Tiwari et al. 2017). Also, a proteomic approach using nLC-Q-TOF analysis of *A. flavus* in response to quercetin treatment showed activation of oxidative stress response proteins and transmembrane transport proteins, which suggests the efficiency of quercetin as a anti-aspergillus phytochemical (Tiwari and Shankar 2018). Therefore, the role of quercetin mediated inhibition of *A. flavus* morphogenesis via calcium signalling and heat-shock protein need further investigations. Recently, SEM and qRT-PCR have been used as efficient tools to determine the phytochemical activity against morphogenetic transformations and expression on various pathways at genomic levels, respectively (Liu et al. 2016; Nishiyama et al. 2017). Thus, the objectives of present study were (i) to determine the effect of quercetin on *A. flavus* conidia at two different time points (4 and 7 h) using scanning electron microscopy; and (ii) to investigate the mechanism of quercetin-mediated inhibition of *A. flavus* germination via genes encoding for calcium signalling pathway and heat-shock protein using qRT-PCR.

## Material and method

### *Growth conditions for* Aspergillus flavus

*Toxigenic Aspergillus flavus* (MTCC 9367), strain which was previously used by Patel et al. (2014) was selected for this study (Patel et al. 2014) and the culture was maintained on potato dextrose agar (potato infusion-20%, dextrose-2%, agar-2%, pH 5.6) slants at 37°C. Spores were harvested after 72 h in phosphate-buffered saline (PBS) with 0.05% tween 20 (PBST) followed by centrifugation at 10,000 rpm (10 min at 4°C). Further, spores were washed with PBS two times and viability check (numbers of CFU/ml) was performed on PDA plates using haemocytometer according to the procedures followed by Tiwari et al. (2016) (Tiwari et al. 2016) and 1 × 10^6^ cells/ml were used as a working conidial culture for this study.

## SEM analysis of quercetin treated *Aspergillus flavus* conidia at 4 and 7 h

For SEM analysis, 1 × 10^6^ conidia of *A. flavus* treated with quercetin at MIC_50_; 113µg/ml and inoculated in sabouraud dextrose broth (dextrose-4%, peptone-1%, pH 5.6) medium against control (*A. flavus* without quercetin) for 4 and 7 h. After that conidia were harvested by centrifugation at 2700 rpm and washed with PBS thrice. Conidia were then fixed in 4% glutaraldehyde in PBS under vacuum for 2–4 h. The cells were washed with distilled water and then the cells were post-fixed with 1% osmium tetroxide for 1 h and dehydrated by passage through increasing concentration of (50–100%) ethanol solutions. The sample was then mounted on an aluminium sheet and coated with gold-palladium alloy. The observations were made on a Zeiss SEM (MA EVO −18 Special Edition).

## Differential gene expression analysis using qRT-PCR

Total RNA from *A. flavus* treated with and without quercetin at 4 and 7 h time points was extracted in two independent biological replicates using the TRIzol method (Invitrogen, USA). Nanodrop spectrophotometer (Thermo Scientific, USA) was used at A_260_/A_280_ nm to estimate the quality and quantity of extracted RNA (Shankar et al. 2011). Further, 1.2% agarose gel electrophoresis was performed to check the integrity of extracted RNA. One micro gram (1 µg) of RNA was used for cDNA synthesis as per manual instructions (Thermo Scientific, USA. Primers for selected *A. flavus* genes were designed using Primer-Blast tool (NCBI) for expression study (Ye et al. 2012) enlisted in Table 1. Further, Bio-Rad machine CFX96 used for qRT-PCR to study the expression of selected genes in tested samples. 100ng of cDNA in12.5 µl reaction was used as a template for RT-PCR using SYBR-Green master-mix (BioRad). From two independent biological replicates, three technical replicates from each were carried for qRT-PCR (Thakur and Shankar 2017). PCR conditions were; initial denaturation at 95°C for 3 min, and 39 cycles of 95°C for 10 s, Tm (52–60) °C for 30 s, 72°C for 45 s. Reference gene *tubulin* was considered for data analysis and melting curve analysis was performed for the replicates for the each gene to check the specificity of primers. In order to calculate the relative expression of genes in samples, ‘2^-∆∆C^_T_” method was used to quantify the expression of selected genes in this study (Livak and Schmittgen 2001)

**Table 1.** List of primers for genes from calcium signalling pathway, tubulin and genes encoding for heat shock proteins.

S. no.	Gene ID *Aspergillus flavus* (NRRL3357)	Gene name	Tm (°C)	Annealing temperature used in PCR Reaction (°C)	Forward and reverse primer sequences
	480538393	Tubulin	55.9	56	5ʹ-GGAATGGATCTGACGGCAAG-3ʹ
			56.8		5ʹ-GGTCAGGAGTTGCAAAGCG-3’
	238487117	cAMP	52.4	52	5ʹ-CTCCACAGGCCCTAATAAC-3ʹ
			52.5		5ʹ-GTGAAGTATCAACGGG-3’
	238496080	rho-gdp	52.2	52	5ʹ-CGAGCTATAAATCCCGAGG-3ʹ
			52.5		5ʹGTCGTTAAGAGGAAGGGTG-3’
	7917368	Pkc	55.1	55	5ʹ-GTAGCGTCTGACTCACAAGG-3ʹ
			55.0		5ʹ-GGCCTTTCGTCCAACCATA-3’
	576867632	Cmd-A	54.7	55	5ʹ-ATCGGTAAGCTTCTCGCC-3ʹ
			54.1		5ʹ-GGATACCGATTCTGAGGAGG-3’
	238498383	Calcium transporting ATPase	53.2	52	5ʹ-AGAACCTATCTCGCTCTCG-3ʹ
			53.5		5ʹ-TCCTGTACTTTCCAGGTCC-3’
	238501501	Crz1	53.5	54	5ʹ-CCACCATCCATTAACGTGG-3ʹ
			53.7		5ʹ-CGGATCAGATTTGCTACGC-3’
	238488300	Plc	52.5	52	5ʹ-GAAGCTCTTAGCAGACTGG-3ʹ
			52.6		5ʹ-GACCGTATGGGTAAATCCG-3’
	238484414	Cal-kinase	55.7	54	5ʹ-GCCATGTCCTAGCTGTGG-3ʹ
			55.2		5ʹ-CCAAGGATACTCTGCATGGG-3’
	62956525	Calcineurin	53.2	52	5ʹ-TACTTCTTCTCGTACCCCG-3ʹ
			53.0		5ʹ-CATCACGCTAGGGAAACC-3’
	238490040	Hsp70	54.5	56	5ʹ-CCTACTCCCTCAAGAACACC-3ʹ
			53.8		5ʹ-GAGACTCGTACTCCTCCTTG-3’
	238484242	Hsp98	52.4	54	5ʹ-GAGAGATGAGGCAGAACG-3ʹ
			53.9		5ʹ-TCCACCTCGAGTCTTTCG-3’
	238503320	Hsp90	55.7	60	5ʹ-CGTCAAGTCCATCACTCAGC-3ʹ
			62.5		5ʹ-GCTTGTGGATGCGCTCGGC-3’
	238493600	Hsp60	53.5	54	5ʹ-GGTTTGACAGCTCCAAGG-3ʹ
			54.2		5ʹ-GTGGTACCAAGGAGAGAGG-3’

Hsp70; Heat shock protein 70, Hsp90; Heat shock protein 90, Hsp60; Heat shock protein 60, Hsp98; Heat shock protein 98, Ca-ATPase; Calcium-transporting ATPase, Cmd-A; Calmodulin, Crz-1;Transcription factor, Ca-Kinase; Calcium Kinase, cAMP-; Cyclic-adenosine monophosphate, Pkc; Protein kinase C, Plc; Phospholipase C, Rho-gdp; Rho family small guanosine diphosphatases

## Statistical analysis

From each independent time point (treated vs control), the significance of the mean values of the data at 4 and 7 h time-point in qRT-PCR was calculated using two-way ANOVA with Bonferronipost test. GraphPad software (GraphPad Prism v 5) was used for the analysis (*** represents *P*-value<0.001).

## Results and discussion

**Figure 1.**
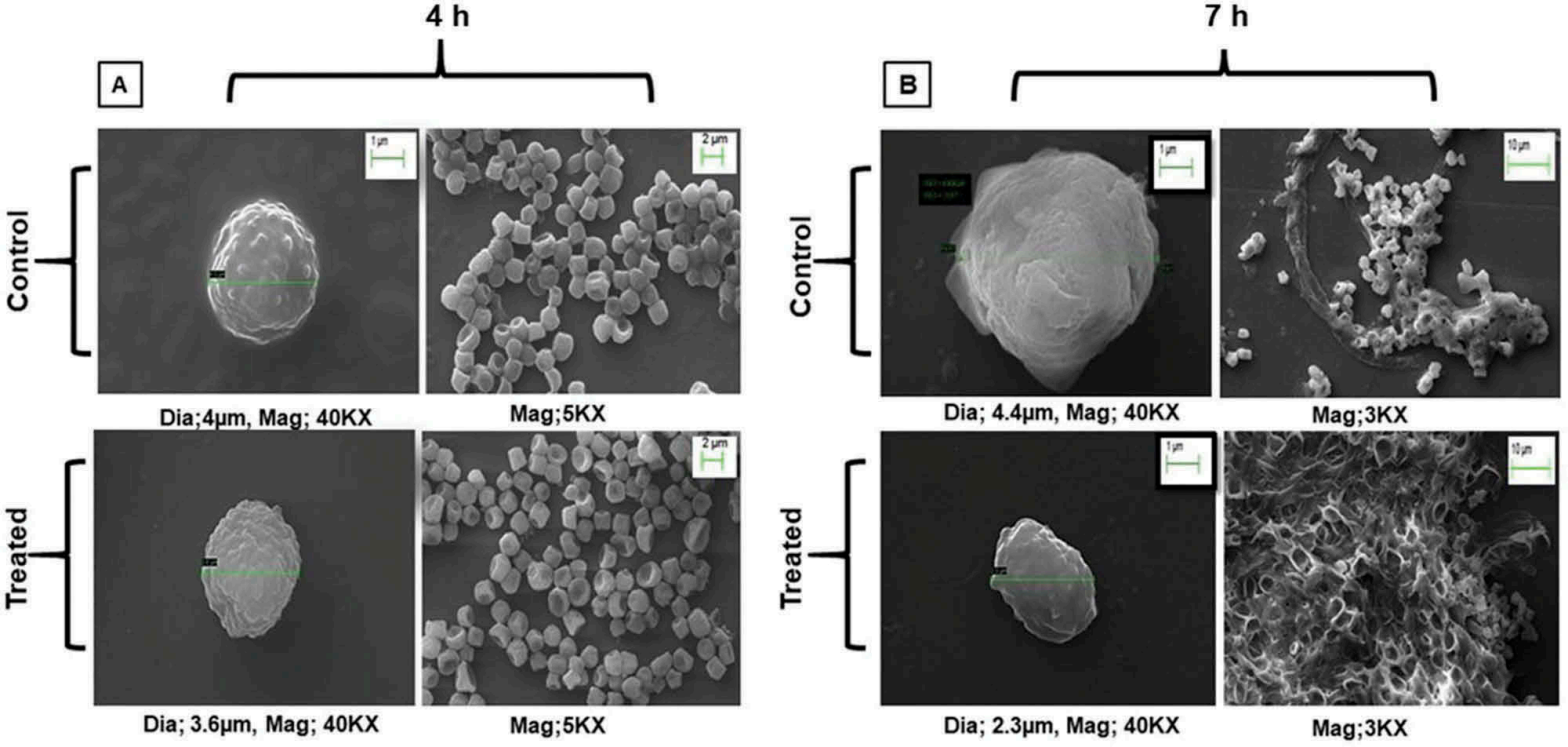
SEM images at different magnifications showing morphological changes in response to quercetin treatment in *A. flavus.*

Germination is the key step in the morphogenesis of conidia, which involves the activation of signalling pathways depending on environmental factors and stress response (Wang et al. 2012). To depict the effect of quercetin on the conidial cell wall of *A. flavus*, SEM analysis was performed at 4 and 7 h to determine the swelling of conidia and isotropic growth. Our study revealed that untreated *A. flavus* conidia were 4 µm in diameter, was found to be more swollen than the quercetin-treated *A. flavus* at 4 h that is 3.6 µm in diameter. Also, at 7 h it has been observed that the size of untreated *A. flavus* conidia was 4.4 µm in comparison to quercetin treated conidia, 2.3 µm. Significant swelling in control *A. flavus* conidia in comparison to quercetin treated conidia were observed (Figure 1). Furthermore, a consistant increase in the size of control conidia from 4 to 7 h was observed in comparison to the treated conidia. Protuberance was observed more in control *A. flavus* conidia during the early stages when compared with the quercetin-treated. Morphogenesis of conidia of *A. flavus* with and without quercetin at 4 and 7 h using SEM have been shown in Figure 1.

Using SEM analysis, we demonstrated that quercetin effectively delayed the morphogenesis of *A. flavus* at two different time points (4 and 7 h). The findings showed that the morphological inhibition starts at the early germination stage of *A. flavus* which increases significantly with time, in response to quercetin. Also, at 4 h, the swelling of conidia and flat-ball shape was not observed in quercetin treated conidia in comparison to control. Whereas at 7 h, less protuberance from the cell surface along with the shrunken and collapsed conidia incapable of polarised hyphal growth were observed in quercetin treated conidia. Further, the SEM studies conducted by Singh et al. (2015) showed apoptotic effects in *C. albicans* in response to quercetin treatment (Singh et al. 2015). Furthermore, in a study by Rauha et al. (2000), quercetin mediated growth inhibition in *A. niger, B. subtilis* and *S. cerevisiae* were observed (Rauha et al. 2000). Overall results showed that quercetin mediated in delaying of swelling as well as isotropic growth of conidia in *A. flavus*.

**Figure 2.**
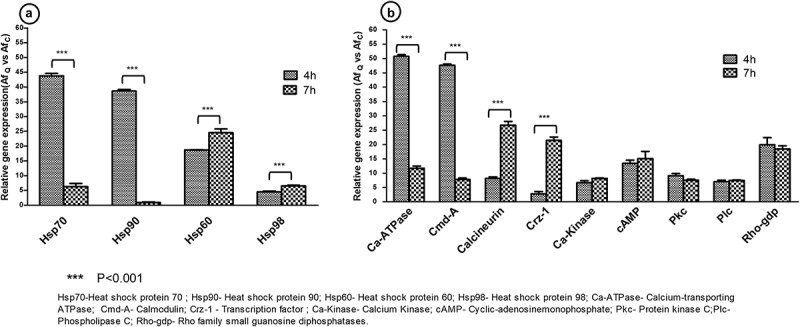
Differential gene expression patterns of heat shock proteins and calcium signalling pathway genes in response to quercetin in *A. flavus*. Panel A illustrated the fold change in expression of genes encoding for heat shock proteins in quercetin treated *A. flavus* in comparison to control at 4 and 7 h time points. Panel B illustrated the fold change in expression of genes related to calcium pathway in quercetin treated *A. flavus* in comparison to control at 4 and 7 h time points. The expression value of each gene was normalised with reference gene “*tubulin*” with their respective time points from treated and control sample. The data represented from the mean of triplicate for each gene from qRT-PCR using two independent biological replicates.

In order to quantify the expression profile of selected genes from calcium signalling pathway in response to quercetin, listed genes in Table 1 were considered. *A. flavus* conidia (with and without quercetin) at 4 and 7 h time points were studied. Up-regulation of genes encoding for Calcium-transporting ATPase (Ca-ATPase) and Calmodulin (*Cmd*) at 4 h time point in quercetin treated sample and also in comparison to 7 h were observed. Whereas, up-regulation of genes encoding for calcineurin and transcription factor (Crz1) were observed at 7 h in treated sample as well as in comparison to 4 h. Other genes such as Ca-kinase, *cAMP, Rho-gdp* and *Pkc* were not significantly modulated with their expression in quercetin treatment in *A. flavus* at 4 and 7 h, however, showed a constitutively higher level of expression in quercetin-treated sample in comparison to control *A. flavus* (Figure 2).

We further report a potential mechanism leading to modulation of Hsp dependent calcium signalling pathway in *A. flavus* in response to quercetin. Calcineurin-Crz1 signalling pathway is the well-established pathway in morphogenesis/dimorphism, fungi virulence and drug resistance (Juvvadi et al. 2017). Activation of calcineurin is mediated by various regulatory pathways which rely on the stress factors which further activates downstream transcriptional factor Crz1 (Juvvadi et al. 2014; Liu et al. 2015b).

Several studies showed that Hsp90 functions synergistically with calcineurin (key mediator) in response to azoles and echinocandins by activating calcium kinases and provided resistance in *C. albicans* (Singh et al. 2009; Gong et al. 2017). Also, Hsp90-calcineurin interaction is important for fungal dimorphism and virulence in fungi such as, *C. albicans, S. cerevisiae* and *P. brasiliensis* (Lee et al. 2013; Tiwari et al. 2015). In response to oxidative stress Hsp90 is also known to activate calmodulin kinases which in turn activates calcineurin-Crz1 pathway (Rodriguez-Caban et al. 2011). In our gene expression study in *A. flavus* under influence of quercetin, we found up-regulation of calcineurin and Crz1 genes at both 4 and 7 h time points, which suggested the activation of calcineurin-Crz1 signalling pathway in response to quercetin-mediated stress. Interestingly, Cramer et al. (2008) using calcineurin and CrzA gene mutant of *A. fumigatus* showed lack of polarised hyphal growth in SEM images (Cramer et al. 2008).

Genes encoding for heat-shock proteins like Hsp60, Hsp70, Hsp90 and Hsp98 were selected for expression analysis with and without quercetin treated (control) in *A. flavus* at two different time points (4 and 7 h). qRT-PCR results showed significant up-regulation of *Hsp70, Hsp90*, at 4 h time point in treated samples in comparison to control and also in comparison to 7 h whereas *Hsp60 and Hsp98* were up-regulated at 7 h in the treated sample and also in comparison to 4 h (Figure 2). Quercetin-mediated response showed up-regulation of both *Hsp70* and *Hsp90* at the early time point (4 h) under phytochemical stress. Up-regulation of *Hsp70* and *Hsp90* at 4 h (in comparison to 7 h and control) suggests that quercetin modulated the expression of Hsp90 potentially to overcome the inhibited swelling of conidia. Other signalling pathway involves the co-operative function of Hsp70 and 90 which activates mitogen-activated protein kinase/protein kinase C (MAPK/PKC) pathway involved in the cell proliferation. Also, Hsp70 and 90 showed a positive interaction with calcineurin in response to drugs (Rodriguez-Caban et al. 2011; Nagao et al. 2012). These findings suggested that under quercetin stress, expression of genes encoding for Hsp70 and Hsp90, and calcineurin-Crz1 signalling pathway are up-regulated to overcome the inhibitory effect of phytochemical. To initiate swelling of conidia, influx of Ca^+2^ in the cytoplasm by calcium-transporting ATPase activates calmodulin, a calcium sensor protein. Calmodulin by activating calcium/calmodulin kinase signals calcineurin pathway (Odom 2014).

On the note, free cytoplasmic Ca^2+^ during polarised growth of fungal hyphae have been observed (Torralba and Heath 2001). In addition, it has been observed that abundant expression of calcium transporter protein, i.e. calcium-transporting ATPase under quercetin treatment in *A. flavus* (Tiwari and Shankar 2018). The effect of quercetin on Ca^+2^ concentration has also been studied using HepG2 cells (Cui et al. 2015) that showed an increase in calcium ion concentration inside the cells and the flow of calcium ions among cell organelles. Whereas in case of breast cell cancer quercetin suggested to induce apoptosis in MDA-MB-231 cells by a reduction in calcium dependent urokinase which leads to decrease in intracellular calcium uptake in cells (Devipriya et al. 2006). Gene transcripts data encoding for calcium transporting ATPase and calmodulin was significantly up-regulated at 4 h (in comparison to 7 h and control) in quercetin treated conidia. These data suggested that quercetin may modulate Ca^+2^ concentrations, and intracellular Ca^+2^ homoeostasis may play an important role during morphogenesis of *A. flavus* conidia.

The next pathway is G-protein coupled receptor pathway which stimulates phospholipase C (*Plc*) involved in the activation of the calcineurin pathway via IP3 (Schumacher et al. 2008). We have observed constitutive higher expression of genes encoding for *cAMP, Pkc, Plc* and *Rho-gdp* at 4 and 7 h in response to quercetin to sustain the swelling and polarised growth of the conidia in both the conditions (Tsai et al. 2013; Tsai and Chung 2014). Overall analysis showed that quercetin modulated the expression of key genes and other elements from signalling pathway.

## Conclusion

Quercetin is a potential flavonoid capable of inhibiting morphogenesis of conidia of *A. flavus*, an essential step in the germination, by modulating the gene expression from calcium signalling pathway, Hsp90 and 70. These findings highlight the potential of calcineurin-Crz signalling pathway for antifungal targets. It also elucidated the role of conserved elements of signal transduction cascades under the phytochemical stress.
